# Treatment of Cardiovascular Dysfunction With PDE5-Inhibitors – Temperature Dependent Effects on Transport and Metabolism of cAMP and cGMP

**DOI:** 10.3389/fphys.2021.695779

**Published:** 2021-07-30

**Authors:** Anders L. Selli, Adrina K. Kuzmiszyn, Natalia Smaglyukova, Timofei V. Kondratiev, Ole-Martin Fuskevåg, Roy A. Lyså, Aina W. Ravna, Torkjel Tveita, Georg Sager, Erik S. Dietrichs

**Affiliations:** ^1^Experimental and Clinical Pharmacology, Department of Medical Biology, UiT – The Arctic University of Norway, Tromsø, Norway; ^2^Division of Surgical Medicine and Intensive Care, University Hospital of North Norway, Tromsø, Norway; ^3^Anesthesia and Critical Care Research Group, Department of Clinical Medicine, UiT – The Arctic University of Norway, Tromsø, Norway; ^4^Department of Laboratory Medicine, Division of Diagnostic Services, University Hospital of North Norway, Tromsø, Norway; ^5^Center for Psychopharmacology, Diakonhjemmet Hospital, Oslo, Norway

**Keywords:** hypothermia, PDE5-inhibitors, cyclic AMP, cyclic GMP, afterload reduction, cardiovascular dysfunction, HAPE, ECMO

## Abstract

**Introduction:**

Cardiovascular dysfunction is a potentially lethal complication of hypothermia. Due to a knowledge gap, pharmacological interventions are not recommended at core temperatures below 30°C. Yet, further cooling is induced in surgical procedures and survival of accidental hypothermia is reported after rewarming from below 15°C, advocating a need for evidence-based treatment guidelines. *In vivo* studies have proposed vasodilation and afterload reduction through arteriole smooth muscle cGMP-elevation as a favorable strategy to prevent cardiovascular dysfunction in hypothermia. Further development of treatment guidelines demand information about temperature-dependent changes in pharmacological effects of clinically relevant vasodilators.

**Materials and Methods:**

Human phosphodiesterase-enzymes and inverted erythrocytes were utilized to evaluate how vasodilators sildenafil and vardenafil affected cellular efflux and enzymatic breakdown of cAMP and cGMP, at 37°C, 34°C, 32°C, 28°C, 24°C, and 20°C. The ability of both drugs to reach their cytosolic site of action was assessed at the same temperatures. IC_50_- and K_*i*_-values were calculated from dose–response curves at all temperatures, to evaluate temperature-dependent effects of both drugs.

**Results:**

Both drugs were able to reach the intracellular space at all hypothermic temperatures, with no reduction compared to normothermia. Sildenafil IC_50_ and K_*i*_-values increased during hypothermia for enzymatic breakdown of both cAMP (IC_50_: 122 ± 18.9 μM at 37°C vs. 269 ± 14.7 μM at 20°C, *p* < 0.05) and cGMP (IC_50_: 0.009 ± 0.000 μM at 37°C vs. 0.024 ± 0.004 μM at 32°C, *p* < 0.05), while no significant changes were detected for vardenafil. Neither of the drugs showed significant hypothermia-induced changes in IC_50_ and K_*i–*_values for inhibition of cellular cAMP and cGMP efflux.

**Conclusion:**

Sildenafil and particularly vardenafil were ableto inhibit elimination of cGMP down to 20°C. As the cellular effects of these drugs can cause afterload reduction, they show potential in treating cardiovascular dysfunction during hypothermia. As in normothermia, both drugs showed higher selectivity for inhibition of cGMP-elimination than cAMP-elimination at low core temperatures, indicating that risk for cardiotoxic side effects is not increased by hypothermia.

## Introduction

Accidental hypothermia is associated with a mortality rate up to 40% and is defined as involuntary drop of body core temperature below 35°C (Vassal et al., 2001). Hypothermia-induced cardiac dysfunction (HCD) contributes to the high mortality (Sessler Daniel, 2001; Kondratiev et al., 2006) and is recognized by decreased cardiac output (CO) as well as increased total peripheral resistance (TPR), during hypothermia and rewarming (Mann et al., 1992; Tveita, 2000; Dietrichs et al., 2015). Similarly, cardiovascular failure is associated with a negative outcome during therapeutic hypothermia (Bush et al., 1995). Other critical cardiovascular conditions, like high altitude pulmonary edema (HAPE), could occur in extreme conditions where exposure to low core temperatures are frequent, and evacuation of patients is difficult (Westensee et al., 2013). In such situations, knowledge about the temperature-dependent effect of relevant pharmacological strategies is paramount, to ensure optimal treatment.

Pharmacological manipulation of intracellular cyclic AMP (cAMP) and cyclic GMP (cGMP) levels is used to influence human cardiovascular function during normothermia. Both cAMP and cGMP are intracellular signal molecules with important function in the cardiovascular system. Intracellular levels of cAMP and cGMP are increased by stimulation of the β-receptor-AC-cAMP-PKA and NO-GC-cGMP-PKG pathways, respectively. Elimination is controlled by enzymatic breakdown and cellular extrusion (Subbotina et al., 2017). Elevated cardiomyocyte cAMP-levels are associated with increased cardiac inotropy and chronotropy, while elevation in peripheral smooth muscle cause relaxation and resulting vasodilation. Elevated levels of cGMP in smooth muscle is also associated with peripheral vasodilation, but in cardiomyocytes it has a slightly negative inotropic effect. cAMP degradation in human cardiomyocytes is largely caused by the phosphodiesterase-3A (PDE3A) enzyme. PDE5A is also present and active both in the healthy and failing human heart, as well as in human blood vessels, and is mainly responsible for cGMP degradation (Johnson et al., 2012). Elimination of cAMP and cGMP is also dependent on the activity of cellular efflux pumps. The ATP-binding cassette subfamily-C 4 (ABCC4) is mainly responsible for transporting cAMP out of cells, while cGMP is thought to be removed by ABCC5 (Jedlitschky et al., 2000; Sellers et al., 2012).

Inotropic effects of well-known adrenergic drugs, such as adrenaline and isoprenaline that elevate cAMP through β-receptor stimulation during normothermia, have been explored in rodent models. Hypothermia induced a paradoxical, negative inotropic effect and increased TPR during hypothermia (Dietrichs et al., 2015, 2016), worsening HCD. Several drugs with a different mechanistic approach to affect the β-receptor-AC-cAMP-PKA and NO-GC-cGMP-PKG pathways during hypothermia and rewarming have therefore been investigated *in vivo* (Tveita and Sieck, 2012; Dietrichs et al., 2014a, 2016; Håheim et al., 2017), with diverging hemodynamic effects. Administration of the potent vasodilator sodium nitroprusside lowered TPR, when administered in hypothermic rats. The results showed a positive effect on CO and prevented HCD (Håheim et al., 2017). Similar results were observed after administration of milrinone, a phosphodiesterase-3 (PDE3) inhibitor, impeding enzymatic breakdown of cAMP. In the hypothermic rat, milrinone-infusion resulted in decreased TPR and increased CO (Dietrichs et al., 2014b).

Total peripheral resistance-reduction therefore appears to be a favorable strategy to prevent HCD (Tveita and Sieck, 2012; Dietrichs et al., 2014a, 2018; Håheim et al., 2017). Elevation of cGMP through PDE5-inhibitors like sildenafil and vardenafil, is a potential pharmacological approach in hypothermic patients and a suggested treatment option in HAPE-patients (Luks et al., 2017). Drug specificity during hypothermic conditions is, however, unknown. Therefore, it is important to investigate the ability of sildenafil and vardenafil to reach their site of action and inhibit cGMP, as well as determining whether they also inhibit cAMP elimination at different stages of hypothermia, encountered in critically ill patients. Exploring the temperature-dependent properties of these clinically relevant drugs, could provide important information on their ability to help sustain cardiovascular functions, during hypothermia and rewarming.

## Materials and Methods

Three different experimental protocols were used to evaluate intracellular access, cellular efflux and phosphodiesterase activity, respectively.

### Temperature

According to temperature-dependent clinical signs and physiological changes, The European Resuscitation Council has classified accidental hypothermia into mild hypothermia (35–32°C), moderate hypothermia (32–28°C) and severe hypothermia (below 28°C) (Truhlar et al., 2015). This classification was used in design of the present experiment, where we collected data at relevant temperatures for both accidental and therapeutic hypothermia. Accordingly, we assessed intracellular access of the drugs and pharmacological inhibition of cAMP- and cGMP-efflux, as well PDE3 and PDE5 at 37°C, 34°C, 32°C, 28°C, 24°C, and 20°C.

### Pharmacological Substances

Sildenafil (Sigma-Aldrich, Schnelldorf, Germany) and vardenafil (Bayer Pharma AG, Wuppertal, Germany) were used in seven different concentrations increasing by a factor of 10, ranging from 1.00E-09 to 1.00E-03 M (1.00 nM to 1.00 mM), to test their potency at both inhibiting elimination of cAMP and cGMP through reducing cellular efflux and enzyme activity.

### Intracellular Access

Both sildenafil and vardenafil are predominantly exerting their effects intracellularly. To estimate whether core temperature reduction would impede their ability to reach the site of action, both drugs were incubated at a concentration of 1.00 μM. The concentration is chosen from therapeutic serum concentrations and were obtained from a systematic literature review in PubMed with (sildenafil) OR (vardenafil) AND (intravenous) AND (plasma concentration) OR (serum concentration) (Table 1). References on intravenous administration was chosen due to patients suffering from hypothermia and HAPE often will have reduced consciousness, making oral administration difficult. Further, bioavailability of oral drugs during hypothermia is hard to predict and oral administration is not an alternative for gaining rapid pharmacological effects. The included reference articles had to report adult human data with a relevant cardiovascular topic. Relevant articles from references were also included. For vardenafil, concentrations from intravenous administration were not available and relevant articles for oral administration were included instead. As the aim for this experiment was to detect potential temperature-dependent effects on access through the cell membrane and potential for free fraction of drugs to increase during hypothermia, we chose a concentration of 1.00 μM for both drugs as this corresponded to the highest serum concentrations after intravenous administration of sildenafil.

**TABLE 1:** Therapeutic plasma concentrations from a literature review of sildenafil andvardenafil, administered for cardiovascular support.

Therapeutic plasma concentration	Calculated concentration in μM	Cardiovascular topic	References	Protein binding	References
Sildenafil	0.449 μM	Pulmonary hypertension	Vachiery et al. (2011)	94–96%	Walker (1999)
	0.101–0.768 μM	Cardiac surgery	Ring et al. (2017)	93–95%	Mehrotra et al. (2007)
Vardenafil	0.010 μM	Pulmonary hypertension	Henrohn et al. (2012)		
	0.044 μM	Pulmonary hypertension	Sandqvist et al. (2013)		

*The search was performed in PubMed with (name of drug) AND (intravenous) AND (plasma concentration) OR (serum concentration). As no articles for intravenous vardenafil administration were found, studies conducted after oral administration were accepted instead.*

Blood was provided by Blodbanken UNN (Department of Immunohematology and Transfusion Medicine, University Hospital of North Norway) where all participants (*n* = 18) were pre-screened and only admitted as donors if they were healthy. Each parallel only included blood from one donor. Experiments were initiated by washing and centrifuging recently (<24 h) drawn EDTA-blood three times with Krebs-Ringer-Phosphate-Buffer containing glucose (KRPB/G, pH∼7.4). The blood was added KRPB/G in a 2.5:1 relationship before measuring hematocrit (Hct) values. Depending on the values, calculations of amount KRPB/G to obtain a Hct of 0.44 were performed, which would later give Hct of 0.40 in the final incubate solution. To start the reaction, 500 μL blood suspension (Hct 0.44) was added to tubes containing 50 μL of either sildenafil, vardenafil or MQ-water (negative control). Each experiment contained triplicates of both drugs and control, and three experiments at each temperature were conducted – in total nine parallels. The reactions were stopped after 30 min (Table 2) by putting the tubes on ice and adding 4 mL ice cold KRPB/G. The reaction solutions were washed and centrifugated three times with ice cold KRPB/G. Fifty microliters of the remaining solution was then added to Eppendorf tubes along with 50 μL internal standard (IS), containing 250 nM IS-Sildenafil-d3 and 500 nM IS-Vardenfail-d5 (Toronto Research Chemicals, ON, Canada). Five samples contained 50 μL known concentrations of sildenafil and vardenafil, and 50 μL IS, and served as controls for accurate analysis. All samples were added 200 mL 0.1 M ZnSO_4_, to lyse the erythrocytes, and then centrifugated. Thirty microliters was taken from Eppendorf tubes for measurements of protein concentration before adding 500 μL acetonitrile. One hundred microliters from each tube was collected for analysis using mass spectrometry (MS).

**TABLE 2:** Experimental protocol.

	Intracellular access			Enzyme inhibition						Cellular efflux inhibition					
Drug	Sildenafil	Vardenafil	Control	Sildenafil		Vardenafil		Control		Sildenafil		Vardenafil		Control	
Target	Human erythrocyte membranes			PDE3	PDE5	PDE3	PDE5	PDE3	PDE5	cAMP-efflux	cGMP-efflux	cAMP-efflux	cGMP-efflux	cAMP-efflux	cGMP-efflux
Incubation time	30 min			30 min						60 min					

Temperature	37°C–34°C–32°C–28°C–24°C–20°C														

*Incubation time of 30 min was chosen for intracellular access and enzyme inhibition, while 60 min was necessary for cellular efflux inhibition to ensure good quality of samples.*

### Cellular Efflux

Cellular extrusion was determined using the inside-out vesicle (IOV) method where erythrocytes from healthy, human donors were sampled. Donors were pre-screened and only admitted as donors by Blodbanken UNN (Department of Immunohematology and Transfusion Medicine, University Hospital of North Norway) if they were healthy. The erythrocytes were separated from plasma by centrifugation and washed. Inside-out vesicles were prepared according to Elin Orvoll et al. (2013) with minor modifications. The membrane vesiculation was initiated by adding hypertonic buffer to the cell suspension. After centrifugation, the suspension was forced through a syringe needle to enhance homogenization of the membranes. IOVs were separated from right side out vesicles (ROV) and unsealed erythrocyte membranes (ghosts) by ultracentrifugation overnight using a density gradient. The uppermost band was collected, washed, and resuspended. Percentage IOV was verified using acetylcholinesterase accessibility test (Ellman et al., 1961). Batches of IOVs used in the parallels were made eight times, including blood from a total of 35 healthy donors.

Inside-out vesicles were then incubated with or without 2 mM ATP and seven different concentrations of sildenafil or vardenafil. The incubation solutions also included radioactive labeled [^3^H]-cGMP and [^3^H]-cAMP (Perkin Elmer, Boston, MA, United States), at a concentration of, respectively, 2 μM and 20 μM. The assays were performed in triplicates at three different days: In total nine parallels were performed to calculate results for each concentration of both drugs at all temperatures. Incubation time of 60 min was chosen to ensure sufficient quality of the samples for each parallel (Table 2). The transport was stopped by adding ice cold buffer. The IOVs were then filtered through a nitrocellulose membrane (Bio-Rad Laboratories, Feldkirchen, Germany), and the membrane was dried. The dried membranes were later added scintillation fluid and radioactivity was measured using a Packard TopCount NXT (Packard, Downers Grove, IL, United States).

Experiments determined total ATP-dependent cellular efflux of cGMP or cAMP from IOVs. As described in previous studies, ABCC5 and ABCC4 are the dominant efflux pumps for cGMP and cAMP (Jedlitschky et al., 2000; Sellers et al., 2012), respectively.

### Phosphodiesterase Activity

Ability of drugs to inhibit cAMP and cGMP hydrolysis by PDE3 and PDE5, respectively, was tested by incubating 5 μM cAMP or cGMP (Sigma-Aldrich, St. Louis, MO, United States) with the seven different concentrations of sildenafil and vardenafil. The assays were performed in triplicates at three different days. A total of nine parallels were used to calculate results for each concentration of both drugs at all temperatures. The reaction was started by adding either a solution containing 0.016 units/μg protein of PDE3 (Abcam, Cambridge United Kingdom), or 0.022 units/μg protein of PDE5 (Sigma-Aldrich, St. Louis, MO, United States), to the Eppendorf tubes. Parallels for cAMP-metabolism included PDE3 only and parallels for cGMP-metabolism included PDE5 only. Control samples were free of drug and was either with or without PDE3 or PDE5. This was done to assure that only the relevant PDE was responsible for breakdown of the cyclic nucleotide, as no other enzyme nor cellular material was added to the incubations. The incubation time was 30 min (see Table 2). Reaction was stopped by adding methanol to the tubes. Internal standard of cGMP/GMP or cAMP/AMP (Sigma-Aldrich, St. Louis, MO, United States, Germany and Toronto Research Chemicals Inc., Toronto, ON, Canada) were added to each sample. Five samples contained only known concentrations of cGMP/GMP or cAMP/AMP and served as calibrators. Samples were analyzed for cGMP/GMP and cAMP/AMP content, using MS.

### Mass Spectrometry (MS) Analysis

Quantification of cAMP/AMP, cGMP/GMP, and PDE5-inhibitors in PDE- and intracellular access experiments were performed with liquid chromatography tandem mass spectrometry (LC-MS/MS). Preparation of samples for LC-MS/MS-analysis is described in the relevant paragraphs above. The method was found to be linear from 0.2 nM to at least 2000 nM (*r*^2^ > 0.998) for cAMP, cGMP, and AMP. For GMP the method was linear from 2 nM to at least 2000 nM (*r*^2^ > 0.998), and 10 nM to at least 5000 nM for the PDE5-inhibitors (*r*^2^ > 0.998). Lower limit of quantification (LLOQ) was found to be 0.2 nM for cAMP, cGMP, and AMP, 2 nM for GMP and 10 nM for the PDE5-inhibitors (2 μl injection volume).

### Data Analysis

The ability of drugs to inhibit cAMP-efflux, cGMP-efflux, PDE3, and PDE5 were determined by calculating IC_50_- and K_*i*_-values from inhibition plots. IC50 values were calculated according to Chou (1976) and data were transformed to K_*i*_-values according to Cheng and Prusoff (1973). IC_25_ and IC_75_ values were estimated by polynomial, cubic regression, based on the inhibition curve of each experiment. Measurement of intracellular concentrations of drugs were adjusted for protein concentrations in each sample. The incubation concentrations were also adjusted for protein concentration in each sample to evaluate the access in percentage. Results are presented as mean ± standard error of mean (SEM). A one-way ANOVA with Holm–Sidak multiple comparisons *post hoc* test was used to compare the IC_50_ and K_*i*_-values for the drugs, as well as intracellular concentrations of drugs, at each temperature with normothermic baseline (37°C). When results were not normally distributed, ANOVA on ranks was used with a Dunn *post hoc* test. Two-tailed *t*-tests were performed to compare IC_50_-values, and adjusted intracellular concentrations, of sildenafil and vardenafil at each temperature. Regression analysis was performed to evaluate whether a linear relationship existed between IC_50_-values and temperature for each drug at each elimination pathway of cAMP and cGMP. Regression analysis was also performed to evaluate if there was a linear relationship between intracellular access of the two drugs and temperature. Pearson’s *r* was calculated for every regression analysis to evaluate how well the calculated lines fitted the observations. *P*-values < 0.05 were considered significant for our data analysis. All analysis were performed using SigmaPlot 14.0 (Systat Software, San Jose, CA, United States, RRID:SCR_003210).

## Results

### Intracellular Access of Drugs During Hypothermia

**FIGURE 1:**
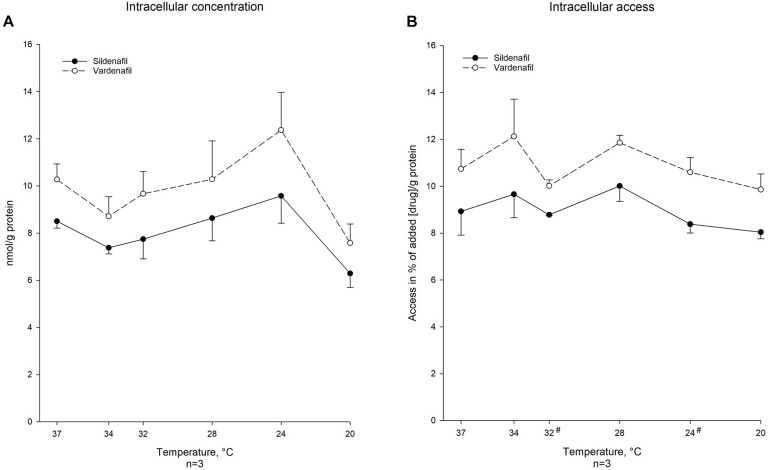
Temperature-dependent intracellular concentration and protein-corrected access in % of added vardenafil and sildenafil. **(A)** Intracellular concentration of sildenafil and vardenafil in nmol/g protein at temperatures ranging from 37°C to 20°C calculated as means ± SEM. Values are calculated from concentration of drug and protein concentration in MS-sample. * Significant difference (*P*-value < 0.05) when nmol/g protein is different from normothermic value. # Significant difference (*P*-value < 0.05) between the two drugs concentrations at specific temperature. **(B)** Intracellular access of sildenafil and vardenafil in % of drug concentration per gram protein in MS-sample compared to drug concentration per gram protein in the incubation solution at temperatures ranging from 37°C to 20°C. Values are in means SEM. * Significant difference (*P*-value < 0.05) when % is different from normothermia. # Significant difference (*P*-value < 0.05) between the two drugs access (in %) at specific temperature.

Sildenafil and vardenafil, at incubate concentration of 1.00 μM, were able to reach the cytosol at all included temperatures from 37°C to 20°C after 30 min incubation (Figure 1). Decreased temperature did not affect the ability of either drug to reach their intracellular site of action. A significantly smaller percentage of sildenafil was able to reach the intracellular space compared to vardenafil at 32°C (% of drug: 8.78 ± 0.027 vs. 10.0 ± 0.257, *p* < 0.05) and at 24°C (% of drug: 8.38 ± 0.381 vs. 10.6 ± 0.625, *p* < 0.05). Regression analysis showed no significant linear relationship between access, neither when looking at total amount nor percentage that reached cytosol, and temperature for any of the drugs (Table 3).

**TABLE 3:** Linear regression analysis for intracellular access and inhibition of phosphodiesterase-5 (PDE5), phosphodiesterase-3 (PDE3), and inhibition of cAMP- and cGMP-efflux calculated at 37°C, 34°C, 32°C, 28°C, 24°C, and 20°C.

Regression analysis	Intracellular access		PDE5-inhibition		PDE3-inhibition		Inhibition of cGMP-efflux		Inhibition of cAMP-efflux	
Drug	Sildenafil	Vardenafil	Sildenafil	Vardenafil	Sildenafil	Vardenafil	Sildenafil	Vardenafil	Sildenafil	Vardenafil
Equation	*y* = 7.14 + 0.063*x*	*y* = 9.14 + 0.059*x*	*y* = 0.064 − 0.002*x*	*y* = 0.039 − 0.001*x*	*y* = 367 − 7.90*x*	*y* = 131 − 3.09*x*	*y* = −2.07 + 19.2*x*	*y* = 9.64 − 0.098*x*	*y* = −7.13 + 0.363*x*	*y* = 26.4 − 0.545*x*
Pearson’s *r*	0.535	0.384	−0.906	−0.921	−0.751	−0.946	0.699	0.224	0.931	0.598
*P*-value	0.21	0.35	<0.01	<0.01	<0.01	<0.01	0.05	0.51	0.13	0.28

*The y-value for the intracellular access equation is added [drug]/g protein and the y-value for inhibition equations are concentration of drug in μM. The x-value is temperature in degrees Celsius for all equation. Pearson’s r is added to depict how well the regression fitted the observations.*

### Cellular Elimination of cGMP

**FIGURE 2:**
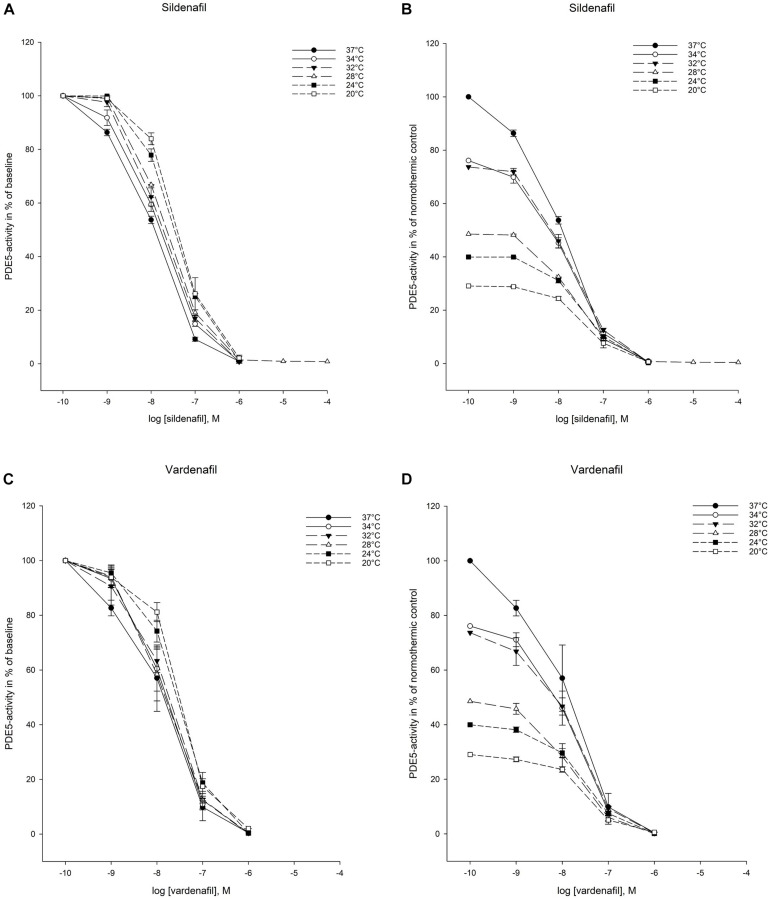
Temperature-dependent inhibition of phosphodiesterase-5 (PDE5) by vardenafil and sildenafi. **(A)** Sildenafil inhibition curves for PDE5-activity at temperatures ranging from 37°C to 20°C. The doses of sildenafil are in logarithm of the concentration in mol/L. **(B)** Inhibition curves for PDE5-activity by sildenafil in % of normothermic inhibition curve at temperatures ranging from 37°C to 20°C. The doses of sildenafil are in logarithm of the concentration in mol/L. **(C)** Vardenafil inhibition curves for PDE5-activity at temperatures ranging from 37°C to 20°C. The doses of vardenafil are in logarithm of the concentration in mol/L. **(D)** Inhibition curves for PDE5-activity by vardenafil in % of normothermic inhibition curve at temperatures ranging from 37°C to 20°C. The doses of vardenafil are in logarithm of the concentration in mol/L.

**FIGURE 3:**
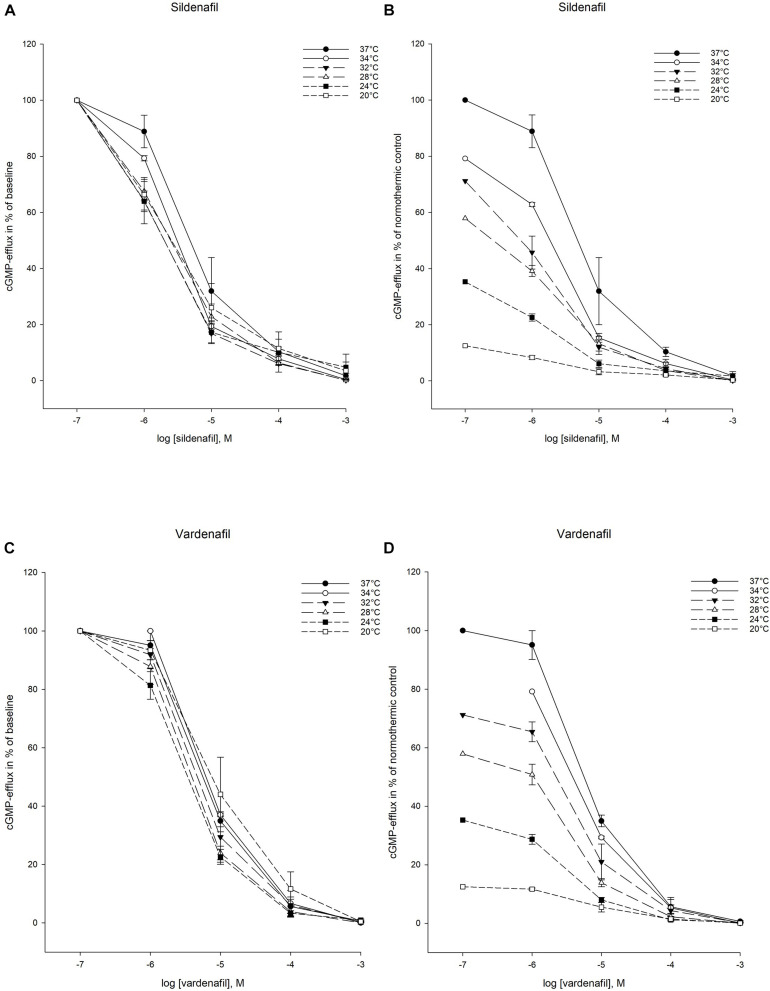
Temperature-dependent inhibition of cGMP-efflux in inside-out vesicles (IOV). **(A)** Sildenafil inhibition curves for ABCC5-activity at temperatures ranging from 37°C to 20°C. The doses of sildenafil are in logarithm of the concentration in mol/L. **(B)** Inhibition curves for ABCC5-activity by sildenafil in % of normothermic inhibition curve at temperatures ranging from 37°C to 20°C. The doses of sildenafil are in logarithm of the concentration in mol/L. **(C)** Vardenafil inhibition curves for ABCC5-activity at temperatures ranging from 37°C to 20°C. The doses of vardenafil are in logarithm of the concentration in mol/L. **(D)** Inhibition curves for ABCC5-activity by vardenafil in % of normothermic inhibition curve at temperatures ranging from 37°C to 20°C. The doses of vardenafil are in logarithm of the concentration in mol/L.

Both sildenafil and vardenafil were able to inhibit cellular elimination of cGMP by PDE5 and efflux pumps at all temperatures (Figures 2, 3).

#### Intracellular Elimination by PDE5

IC_50_- and K_*i*_ -values for PDE5 inhibition were significantly increased compared to normothermic baseline at 32°C for sildenafil (IC_50_: 0.024 ± 0.004 vs. 0.009 ± 0.000 μM, *p* < 0.05) and remained elevated down to 20°C, with exception of measurements at 28°C (Tables 4, 5). For vardenafil, IC_50_-values did not significantly differ from normothermic control at any temperatures. When comparing IC_50_- and K_*i*_-values of sildenafil and vardenafil at different temperatures, sildenafil had significantly higher IC_50_- and K_*i*_ -values at 20°C (IC_50_: 0.037 ± 0.003 vs. 0.023 ± 0.002 μM, *p* < 0.05). The IC_25_–IC_75_ interval was significantly increased for sildenafil compared to normothermic baseline at 24°C (IC_25_–IC_75_: 0.098 ± 0.012 μM vs. 0.038 ± 0.000 μM, *p* < 0.05) and 20°C (IC_25_–IC_75_: 0.132 ± 0.048 μM 20°C vs. 0.038 ± 0.000 μM, *p* < 0.05) (Table 6).

**TABLE 4:** IC_50_-values for inhibition of phosphodiesterase-5 (PDE5), phosphodiesterase-3 (PDE3), and inhibition of cAMP- and cGMP-efflux at temperatures ranging from 37°C to 20°C.

IC_50_	PDE5		PDE3		cGMP-efflux		cAMP-efflux	
Temperature	Sildenafil (μM)	Vardenafil (μM)	Sildenafil (μM)	Vardenafil (μM)	Sildenafil (μM)	Vardenafil (μM)	Sildenafil (μM)	Vardenafil (μM)
37°C	0.009 ± 0.000	0.008 ± 0.003	122 ± 18.9^#^	16.5 ± 6.08^#^	6.96 ± 2.58	5.26 ± 1.46	5.79 ± 1.73	5.85 ± 0.51
34°C	0.014 ± 0.002	0.015 ± 0.005	91.9 ± 12.6^#^	24.9 ± 5.73^#^	3.68 ± 0.416^#^	9.40 ± 0.762^#^	5.91 ± 2.22	5.82 ± 0.685
32°C	0.024 ± 0.004*	0.015 ± 0.004	103 ± 14.1^#^	40.5 ± 3.78^#^	2.53 ± 0.878	6.50 ± 1.20	4.66 ± 1.37^#^	13.0 ± 1.69^#^
28°C	0.017 ± 0.003	0.015 ± 0.003	95.7 ± 16.1^#^	32.7 ± 11.0^#^	2.98 ± 0.304	4.94 ± 0.891	2.73 ± 0.382	9.63 ± 5.79
24°C	0.028 ± 0.005*	0.022 ± 0.003	143 ± 44.8	55.5 ± 4.75	2.21 ± 0.434	3.75 ± 0.926	−	−
20°C	0.037 ± 0.003*^#^	0.023 ± 0.002^#^	269 ± 14.7*^#^	73.4 ± 26.3^#^	2.76 ± 1.27	10.9 ± 4.79	−	−

Values are mean ± SEM and given in ±M. *Significant difference (P-value < 0.05), when compared to normothermic control. ^#^Significant difference (P-value < 0.05) when IC_50_ values of sildenafil and vardenafil are compared at same temperature. Neither of the drugs inhibited ABCC4-activity below 28°C.

**TABLE 5:** K_*i*_-values for inhibition of phosphodiesterase-5 (PDE5), phosphodiesterase-3 (PDE3), and inhibition of cAMP- and cGMP-efflux at temperatures ranging from 37°C to 20°C.

K_*i*_	PDE5-inhibition		PDE3-inhibition		cGMP-efflux		cAMP-efflux	
Temperature	Sildenafil (μM)	Vardenafil (μM)	Sildenafil (μM)	Vardenafil (μM)	Sildenafil (μM)	Vardenafil (μM)	Sildenafil (μM)	Vardenafil (μM)
37°C	0.002 ± 0.000	0.002 ± 0.001	5.61 ± 0.865^#^	0.757 ± 0.276^#^	3.93 ± 1.46	2.97 ± 0.823	3.51 ± 1.05	3.55 ± 0.311
34°C	0.004 ± 0.000	0.004 ± 0.001	4.21 ± 0.579^#^	1.14 ± 0.263^#^	2.08 ± 0.235^#^	5.31 ± 0.431^#^	3.59 ± 1.35	3.53 ± 0.415
32°C	0.006 ± 0.001*	0.004 ± 0.001	4.66 ± 0.647^#^	1.85 ± 0.173^#^	1.43 ± 0.497	3.67 ± 1.13	2.83 ± 0.828^#^	7.86 ± 1.02^#^
28°C	0.004 ± 0.001	0.004 ± 0.001	4.38 ± 0.739^#^	1.50 ± 0.505^#^	1.68 ± 0.172	2.79 ± 0.504	1.66 ± 0.232	5.84 ± 3.51
24°C	0.007 ± 0.001*	0.006 ± 0.001	6.49 ± 2.05	2.54 ± 0.218	1.25 ± 0.245	2.12 ± 0.523	−	−
20°C	0.009 ± 0.001*^#^	0.006 ± 0.000^#^	12.3 ± 0.675*^#^	3.36 ± 1.21^#^	1.56 ± 0.718	6.17 ± 2.71	−	−

Values are mean ± SEM and given in μM. *Significant difference (P-value < 0.05) when compared to normothermic control. ^#^Significant difference (P-value < 0.05) when IC50 values of sildenafil and vardenafil are compared at same temperature. Neither of the drugs inhibited cAMP-efflux below 28°C.

**TABLE 6:** IC_25_–IC_75_ intervals, describing the concentration needed to increase inhibition of phosphodiesterase-5 (PDE5), phosphodiesterase-3 (PDE3), and inhibition of cAMP- and cGMP-efflux from 25% to 75%, at temperatures ranging from 37°C to 20°C.

IC_25_–IC_75_	PDE5		PDE3		cGMP-efflux		cAMP-efflux	
Temperature	Sildenafil (μM)	Vardenafil (μM)	Sildenafil (μM)	Vardenafil (μM)	Sildenafil (μM)	Vardenafil (μM)	Sildenafil (μM)	Vardenafil (μM)
37°C	0.038 ± 0.000	0.059 ± 0.029	460 ± 38.8	61.0 ± 14.3	23.6 ± 9.08	12.6 ± 3.89	27.1 ± 9.36	30.9 ± 6.82
34°C	0.055 ± 0.004	0.028 ± 0.012	411 ± 40.4	72.1 ± 3.24	13.5 ± 1.74	19.2 ± 3.16	19.8 ± 1.82	19.9 ± 0.967
32°C	0.060 ± 0.006	0.062 ± 0.016	485 ± 32.4	107 ± 27.0	9.76 ± 3.99	18.0 ± 4.12	20.0 ± 4.27	27.3 ± 6.19
28°C	0.083 ± 0.008	0.049 ± 0.016	450 ± 35.9	128 ± 35.9	12.0 ± 1.96	10.6 ± 2.75	13.8 ± 3.85	17.3 ± 6.59
24°C	0.098 ± 0.012*	0.074 ± 0.005	584 ± 55.0	148 ± 31.9	11.4 ± 4.73	13.0 ± 1.74	−	−
20°C	0.132 ± 0.048*	0.103 ± 0.027	697 ± 22.5*	709 ± 105*	19.2 ± 8.45	28.1 ± 14.6	−	−

Values are mean ± SEM given in μM. *Significant difference (P-value < 0.05) when compared to normothermic control. Neither of the drugs inhibited cAMP-efflux below 28°C.

The temperature-dependent increase in IC_50_ for both sildenafil and vardenafil appeared to follow a linear pattern (Table 3). Regression analysis provided the equation for calculating sildenafil IC_50_ values during hypothermia: *y* = 0.064 − 0.002*x* μM, with *x* being the temperature in Celsius. The *R*-value was −0.906 (*p* < 0.05). For vardenafil the calculated equation was *y* = 0.039 − 0.001*x* μM, with *R* = −0.921 (*p* < 0.01).

#### Cellular Efflux

For ABBC5 inhibition, there were no statistically significant difference in IC_50_- and K_*i*_-values for either sildenafil or vardenafil when compared to 37°C (Tables 4, 5). Only at 32°C there was a significant lower IC_50_- and K_*i*_-value for sildenafil than vardenafil (IC_50_: 3.68 ± 0.416 vs. 9.40 ± 0.762 μM, *p* < 0.05). The IC_25_–IC_75_ interval remained stable for both drugs during hypothermia.

Regression analysis showed that neither sildenafil nor vardenafil inhibition of cGMP-efflux followed a linear pattern during temperature reduction (Table 3).

### Cellular Elimination of cAMP

**FIGURE 4:**
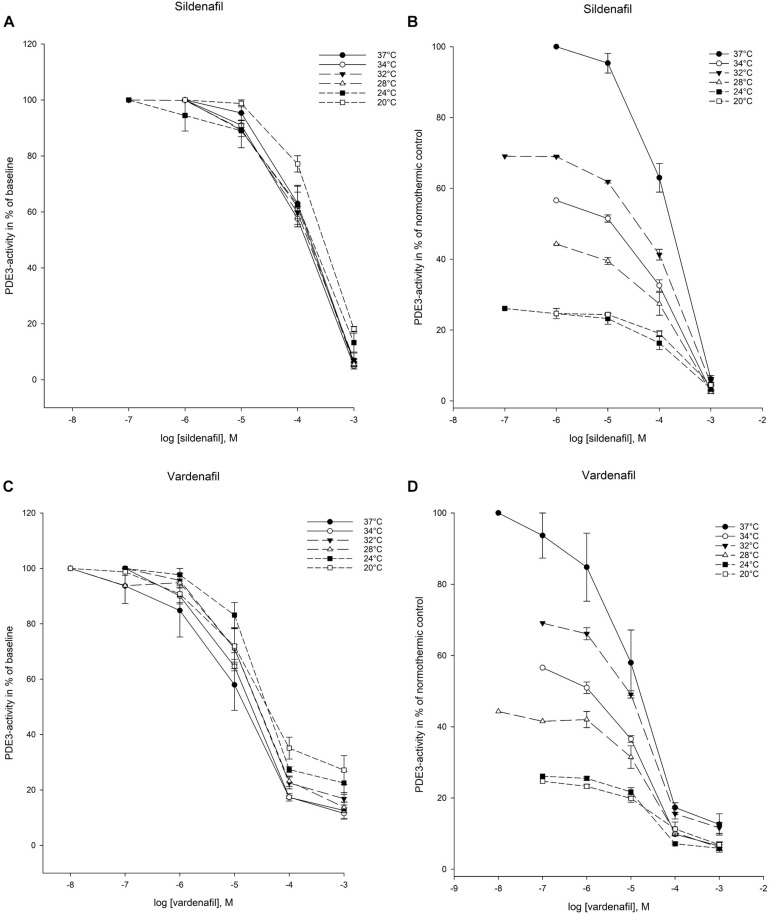
Temperature-dependent inhibition of phosphodiesterase-3 (PDE3) by vardenafil and sildenafil. **(A)** Sildenafil inhibition curves for PDE3-activity at temperatures ranging from 37°C to 20°C. The doses of sildenafil are in logarithm of the concentration in mol/L. **(B)** Inhibition curves for PDE3-activity by sildenafil in % of normothermic inhibition curve at temperatures ranging from 37°C to 20°C. The doses of sildenafil are in logarithm of the concentration in mol/L. **(C)** Vardenafil inhibition curves for PDE3-activity at temperatures ranging from 37°C to 20°C. The doses of vardenafil are in logarithm of the concentration in mol/L. **(D)** Inhibition curves for PDE3-activity by vardenafil in % of normothermic inhibition curve at temperatures ranging from 37°C to 20°C. The doses of vardenafil are in logarithm of the concentration in mol/L.

**FIGURE 5:**
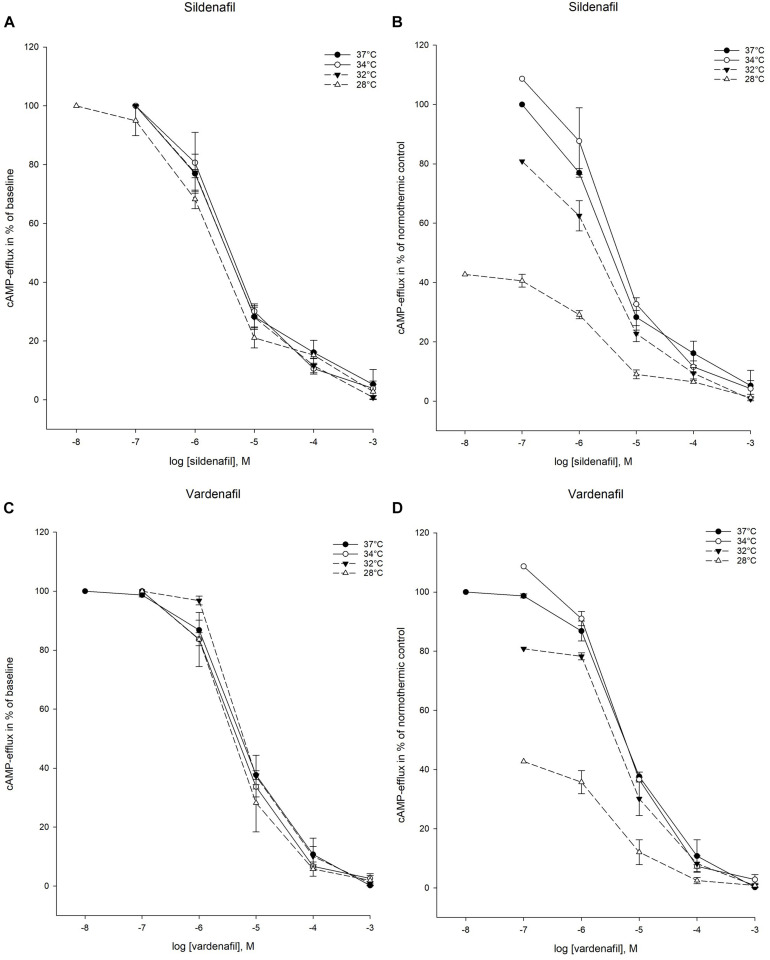
Temperature-dependent inhibition of cAMP-efflux in inside-out vesicles (IOV). **(A)** Sildenafil inhibition curves for ABCC4-activity at temperatures ranging from 37°C to 28°C. The doses of sildenafil are in logarithm of the concentration in mol/L. **(B)** Inhibition curves for ABCC4-activity by sildenafil in % of normothermic inhibition curve at temperatures ranging from 37°C to 28°C. The doses of sildenafil are in logarithm of the concentration in mol/L. **(C)** Vardenafil inhibition curves for ABCC4-activity at temperatures ranging from 37°C to 28°C. The doses of vardenafil are in logarithm of the concentration in mol/L. **(D)** Inhibition curves for ABCC4-activity by vardenafil in % of normothermic inhibition curve at temperatures ranging from 37°C to 28°C. The doses of vardenafil are in logarithm of the concentration in mol/L.

PDE3-mediated elimination of cAMP was inhibited by both sildenafil and vardenafil at all temperatures in the experimental protocol (Figure 4). Inhibition of cAMP-efflux was however only achieved down to 28°C (Figure 5). At the two lowest temperatures, 24°C and 20°C, neither of the drugs were able to inhibit cAMP-efflux. These temperatures are therefore excluded from IC_50_ and K_*i*_ calculations.

#### Intracellular Elimination by PDE3

IC_50_- and K_*i*_-values for PDE3-inhibition was substantially increased for sildenafil at 20°C compared to normothermic control (IC_50_: 269 ± 14.7 vs. 122 ± 18.9 μM, *p* < 0.05). For vardenafil, there were no significant differences compared to normothermia. Differences in IC_50_- and K_*i*_-values between drugs, *p* < 0.05, were observed at all temperatures except 24°C (Tables 4, 5). The IC_25_–IC_75_ interval was significantly increased for both sildenafil and vardenafil compared to normothermic baseline at 20°C (IC_25_–IC_75_ sildenafil: 697 ± 22.5 μM vs. 460 ± 38.8 μM, *p* < 0.05. IC_25_–IC_75_ vardenafil: 790 ± 105 μM vs. 61.0 ± 14.3 μM, *p* < 0.05) (Table 6).

Regression analysis showed significant correlation (*p* < 0.05) between IC_50_ values and temperature for sildenafil with *y* = 367 − 7.90*x* μM, *R* = −0.751. For vardenafil, there was a significant correlation (*p* < 0.05) with *R* = −0.946, providing the equation *y* = 131 – 3.09*x* μM (Table 3).

#### Cellular Efflux

The IC_50_- and K_*i*_-values for inhibition of cAMP-efflux by sildenafil and vardenafil at hypothermic temperatures, showed no significant difference when compared to normothermic control. When comparing drugs however, IC_50_- and K_*i*_-values for sildenafil were significantly lower than vardenafil at 32°C (IC_50_: 4.66 ± 1.37 vs. 13.0 ± 1.69 μM, *p* < 0.05) (Tables 4, 5). The IC_25_–IC_75_ interval remained stable for both drugs during hypothermia.

Regression analysis showed no significant correlation for IC_50_ pattern with temperature for neither of the drugs (Table 3).

### Drug Selectivity

#### Phosphodiesterase Enzymes

As expected, calculated ratios between IC_50_ values showed that the sildenafil concentration needed to inhibit PDE3 was higher (by a factor of 14400) than the dose needed to achieve PDE5-inhibition during normothermic conditions (37°C). Vardenafil was less selective, with a PDE3/PDE5-inhibiting ratio of 1960. Hypothermia appeared to reduce PDE5-selectivity of sildenafil, with a PDE3/PDE5-inhibiting ratio of 7270 at 20°C and increase PDE5-selectivity of vardenafil, as the PDE3/PDE5-inhibiting ratio was increased to 3230 at 20°C (Table 7).

**TABLE 7:** Drug selectivity for sildenafil and vardenafil at temperatures ranging from 37°C to 20°C.

Drug selectivity [IC_50_]/[IC_50_]	[PDE3-inhibition]/[PDE5-inhibition]		[cAMP-efflux-inhibition]/[cGMP-efflux-inhibition]		[cGMP-efflux-inhibition]/[PDE5-inhibition]		[cAMP-efflux-inhibition]/[PDE3-inhibition]	
				
Temperature	Sildenafil	Vardenafil	Sildenafil	Vardenafil	Sildenafil	Vardenafil	Sildenafil	Vardenafil
37°C	14400	1960	0.832	1.11	815	625	0.047	0.354
34°C	6730	1650	1.61	0.620	269	622	0.064	0.234
32°C	4240	2630	1.84	1.99	105	422	0.046	0.320
28°C	5780	2220	0.917	1.95	180	335	0.029	0.294
24°C	5110	2510	−	−	79.7	170	−	−
20°C	7270	3230	−	−	74.5	480	−	−

*Values are ratios between IC_50_-values for different elimination ways of cAMP and cGMP. Neither of the drugs inhibited cAMP-efflux below 28°C.*

#### Efflux Pumps

The normothermic cAMP-efflux/cGMP-efflux IC_50_-ratio for sildenafil (0.832) and vardenafil (1.11) indicate that both drugs inhibit cAMP and cGMP efflux at similar concentrations. The tendency for both drugs is a modest increase in selectivity for cGMP-efflux during hypothermia, with a sildenafil-ratio of 0.917 and vardenafil-ratio of 1.95 at 20°C (Table 7).

#### cGMP Elimination

During normothermic conditions, the predominant inhibition of cGMP-elimination by sildenafil and vardenafil, is through PDE5-inhibition, as the cGMP-efflux/PDE5 inhibition-ratio was 815 and 625, respectively. For both drugs the ratios decreased during hypothermia to 170 for vardenafil at 24°C and 74.5 for sildenafil at 20°C (Table 7).

#### cAMP Elimination

Both sildenafil and vardenafil are more efficient inhibitors of cAMP efflux than PDE3-mediated elimination in normothermic conditions, with an cAMP-efflux/PDE3 inhibition-ratio of 0.047 for sildenafil and 0.354 for vardenafil. Values decreased with reduced temperature. At 28°C the inhibition-ratios for sildenafil was 0.029, and 0.294 for vardenafil (Table 7).

## Discussion

Temperature reduction does not affect the ability of PDE5-inhibitors sildenafil and vardenafil to reach their cytosolic site of action. Our results show that both drugs inhibit elimination of cGMP, as well as cAMP, at temperatures down to 20°C. The concentration of sildenafil needed to inhibit PDE-enzymes is increased with temperature reduction, while there is a tendency toward increased sensitivity of efflux pumps to sildenafil-inhibition. Vardenafil concentrations needed to inhibit all elimination pathways remain unchanged during temperature reduction. Establishing the pharmacodynamic properties of these PDE5-inhibitors is crucial in the process of developing better treatment-guidelines for cardiovascular complications in accidental and therapeutic hypothermia.

Accidental hypothermia guidelines recommend to avoid pharmacological treatment until a body core temperature of minimum 30°C is reached (No Author, 2000; Truhlar et al., 2015). Subsequently, use of vasopressors are listed as the preferred drugs for cardiovascular support, despite the benefits being unclear (Paal et al., 2016). There are indications that this strategy might be unfavorable. In experimental hypothermia and rewarming, increased afterload is associated with HCD and a poor outcome (Dietrichs et al., 2014a, 2015; Håheim et al., 2017). Contradictory to current recommendations, pharmacologically induced vasodilation emerge as a promising strategy to prevent HCD and elevate organ blood flood during rewarming (Håheim et al., 2017, 2020). cGMP elevation is a central mechanism for vasodilation that remains intact during severe hypothermia and rewarming (Håheim et al., 2017, 2020). Pharmacological cGMP-increase is achieved either through stimulating intracellular production, or through reducing elimination. At low core temperatures, it is apparent that drug-induced cGMP production, through stimulating the NO-GC-cGMP-PKG-pathway, is a challenging strategy. Doses of nitroprusside giving favorable effects in normothermia proved harmful in severe hypothermia, due to elevated potency, while doses adjusted to effect on mean arterial pressure (MAP) reduction were favorable (Håheim et al., 2017). In this context, elevating intracellular cGMP through inhibiting elimination, could prove a physiological approach to achieve reduction of afterload, without causing uncontrolled reduction of MAP.

Our findings demonstrate that the difference between IC_50_-values for cGMP-efflux-inhibition and PDE5-inhibition decreases during cooling, indicating that the elimination of cGMP during hypothermia is more dependent on cGMP-efflux. IC_25_–IC_75_ intervals were calculated to estimate a pharmacodynamic window of effect for both drugs on all elimination pathways. No decrease was detected during hypothermia. Targeting cGMP-efflux, in addition to PDE5, therefore appears to be a relevant strategy for cardiovascular support during rewarming from hypothermia. We show that sildenafil and vardenafil are both able to inhibit these cGMP-elimination pathways at low core temperatures, and that they therefore show potential for treatment of hypothermic patients.

There is consensus of venoarterial extra corporeal membrane oxygenation (VA-ECMO) being the preferred treatment of hemodynamically unstable hypothermic patients (No Author, 2000; Brown et al., 2012; Truhlar et al., 2015; Saczkowski et al., 2018). During such treatment, cardiovascular complications are common (Rao et al., 2018; Choi et al., 2019). Left ventricle (LV) dysfunction can appear as not all of the circulating blood is directed through the VA-ECMO device. Some will still pass through the pulmonary circulation. Since MAP is increased by VA-ECMO, the LV has to overcome increased afterload to maintain ejection fraction. Failure could lead to LV-distention and elevated pressure, with a backward failure giving increased pulmonary pressure and edema (Lo Coco et al., 2018; Rao et al., 2018). Risk is higher if the patient has an underlying LV-dysfunction, like HCD (Dietrichs et al., 2018). Pharmacological afterload reduction is a suggested treatment strategy to alleviate backward failure during VA-ECMO-treatment, but it is important to avoid systemic hypotension (Rao et al., 2018). In the present experiment, inhibition of cGMP-efflux is achieved at supratherapeutic concentrations during both normothermia and hypothermia, while PDE5 inhibition by sildenafil or vardenafil, appears a promising strategy to achieve physiologically balanced afterload reduction, and prevent pulmonary edema in hypothermic VA-ECMO patients (Lo Coco et al., 2018).

In addition to afterload reduction, inotropic support could also be favorable during VA-ECMO-treatment (Rao et al., 2018). Inotropes are administered to help overcome the increased afterload and maintain LV ejection fraction (Rao et al., 2018). We show that sildenafil and vardenafil inhibit elimination of cAMP during hypothermia. Earlier studies have shown positive inotropic effect of PDE3-inhibition *in vivo* (Dietrichs et al., 2014a, 2018). IC_50_-values for PDE5-inhibition by sildenafil and vardenafil are in the nM-range, while IC_50_-values for PDE3 and inhibition of cAMP-efflux are in the μM-range. These concentrations are supratherapeutic during normothermia (Tables 4, 5). In order to provide evidence-based inotropic support during VA-ECMO-treatment in hypothermic patients, further studies on drugs that target inhibition of cAMP-elimination are needed as sildenafil and vardenafil appears to be ineffective in therapeutic doses.

Treatment of hemodynamically unstable hypothermic patients, face some of the same challenges as pre-hospital HAPE-treatment, when evacuation is difficult, and the patient is exposed to low temperatures. Sildenafil has been proposed as treatment, as it can be administered when oxygen treatment and rapid decent is impossible (Bates et al., 2007). The strong linear correlation between IC_50_-value and decreasing temperature may serve as a helpful tool in low ambient temperatures, as effect is decided according to measured core temperature. Further investigation of pharmacokinetic data could complement this finding and help develop evidence-based guidelines, with pinpointed dose recommendations for cardiovascular support in hypothermic patients. Although sildenafil and tadalafil are the only PDE5-inhibitors suggested in the treatment of HAPE (Maggiorini et al., 2006; Bates et al., 2007), vardenafil may now be suggested as a candidate drug. Our findings show little pharmacodynamic change, meaning that clinicians only need to account for the impact of hypothermia on pharmacokinetic properties, when calculating adequate vardenafil dosage.

Metabolism of sildenafil and vardenafil is performed in the liver mainly by CYP3A4 but also CYP2C9 and CYP3A5 (Huang and Lie, 2013). Enzyme affinity is decreased with reduction of core temperature, impeding elimination (Tortorici et al., 2006; van den Broek et al., 2010). CYP3A4, the main metabolizing enzyme of both sildenafil and vardenafil is shown to have an activity of 48% at 26°C and 68% at 32°C (Fritz et al., 2005). Sildenafil and vardenafil are both metabolized to active metabolites that are less potent than the parent compounds (Hyland et al., 2001; Bischoff, 2004b). These metabolites are largely eliminated through biliary excretion (Mehrotra et al., 2007), which also is impaired during hypothermia (van den Broek et al., 2010). Decreased activity of CYP-enzymes and biliary excretion during hypothermia, alongside changed plasma protein binding (van den Broek et al., 2010), will lead to slower metabolism of the PDE5-inhibitors, slower production of active metabolites, reduced excretion, altered free fraction and thus, unpredictable therapeutic effect and increased risk of toxicity. In order to safely introduce the use of PDE5-inhibitors in treatment of cardiovascular complications during hypothermia and rewarming, these pharmacokinetic aspects need to be addressed through further experiments.

Although PDE3 and PDE5 are the main targets for pharmacological agents aiming to treat cardiovascular conditions through PDE-inhibition, other PDEs could also be affected by sildenafil and vardenafil administration. None have been investigated during hypothermia. In therapeutic concentrations of the PDE5-inhibitors, the isoenzyme closest in IC_50_-value, and of cardiovascular relevance, is PDE1 (Bischoff, 2004a; Levy et al., 2011). PDE2, PDE4, PDE6 and PDE9 may also influence cardiovascular functions by inhibition of metabolism of cAMP, cGMP or both. However, these PDEs have much higher IC_50_-values for sildenafil and vardenafil during normothermia (Adderley et al., 2010; Kim and Kass, 2017). Studies on other PDEs were excluded from our experiment due to PDE5 being the main target for afterload reduction by vascular smooth muscle and PDE3 being the target for inotropic support in cardiac muscle. Our results show that sildenafil and vardenafil largely remain specific for PDE5. Further studies looking at other relevant PDEs in our hypothermic model would provide a better overall description on possible inhibition of other PDEs by sildenafil and vardenafil. PDEs are also known to interact when present in the same tissue or experimental solutions (Zhao et al., 2015). Assessing these different aspects could provide more information about potential effects and side effects of the drugs during hypothermia and therefore remains to be studied in future studies in our model.

## Conclusion

Sildenafil and vardenafil are able to reach cytosol and IC_50_-values for cGMP-elimination remain intact or predictable at temperatures down to 20°C. As the cellular effects of these drugs can cause afterload reduction, they show potential in treating cardiovascular dysfunction during hypothermia. Our findings lay foundation for *in vivo* studies and further development of evidence-based, pharmacological treatment guidelines in both accidental and therapeutic hypothermia, as well as in HAPE-patients.

## Data Availability Statement

The raw data supporting the conclusions of this article will be made available by the authors, without undue reservation.

## Ethics Statement

Ethical review and approval was not required for the study on human participants in accordance with the local legislation and institutional requirements. The patients/participants provided their written informed consent to participate in this study.

## Author Contributions

AS, AK, NS, and TK conducted the experiments in the lab. RL helped with technical issues and theoretical questions during experiments. O-MF analyzed the results using mass-spectrometry. AS and ED interpreted the results and performed the statistics. AR, TT, GS, and ED planned the research project. AS, ED, O-MF, NS, and GS contributed to the manuscript. All authors read and approved the manuscript.

## Conflict of Interest

The authors declare that the research was conducted in the absence of any commercial or financial relationships that could be construed as a potential conflict of interest.

## Publisher’s Note

All claims expressed in this article are solely those of the authors and do not necessarily represent those of their affiliated organizations, or those of the publisher, the editors and the reviewers. Any product that may be evaluated in this article, or claim that may be made by its manufacturer, is not guaranteed or endorsed by the publisher.
